# Efficacy of artesunate-amodiaquine for the treatment of acute uncomplicated falciparum malaria in southern Mauritania

**DOI:** 10.1186/1475-2875-13-496

**Published:** 2014-12-16

**Authors:** Mohamed Ouldabdallahi, Ismail Alew, Mohamed Salem Ould Ahmedou Salem, Mamadou dit Dialaw Ba, Ali Ould Mohamed Salem Boukhary, Mohamed Lemine Ould Khairy, Mohamed Boubacar Abdel Aziz, Pascal Ringwald, Leonardo K Basco, Saidou Doro Niang, Sid Mohamed Lebatt

**Affiliations:** Unité de Recherche « Génsome et Milieux », Faculté des Sciences et Techniques, Université des Sciences, de Technologie et de Médecine, Nouakchott, Mauritania; Initiative mauritanienne pour la lutte contre les maladies endémiques « MEDCINGO », ilôt 358, Riyad Pk8, Nouakchott, Mauritania; Ministère de la Santé, Nouakchott, Mauritania; World Health Organization, Ilot K140-141, Route de la corniche ouest, Tevragh Zeina, Nouakchott BP 320 Mauritania; Drug Resistance and Containment, Global Malaria Programme, World Health Organization, 20 Avenue Appia, 1211 Geneva 27, Switzerland; Unité Mixte de Recherche 198, Unité de Recherche sur les Maladies Infectieuses et Tropicales Emergentes (URMITE), Institut de Recherche pour le Développement, Faculté de Médecine La Timone, Aix-Marseille Université, Marseille, France

**Keywords:** Drug resistance, Plasmodium falciparum, Artemisinin, 4-aminoquinolines, Clinical trial

## Abstract

**Background:**

A regular evaluation of therapeutic efficacy in sentinel sites and a system of surveillance are required to establish treatment guidelines and adapt national anti-malarial drug policy to the rapidly changing epidemiology of drug-resistant malaria. The current anti-malarial treatment guideline in Mauritania, officially recommended since 2006, is based on artemisinin-based combination therapy. The aim of the present study was to evaluate clinical efficacy and tolerance of artesunate-amodiaquine, the first-line treatment for acute uncomplicated malaria, in Mauritanian paediatric and adult patients to validate its continued use in the country.

**Methods:**

*Plasmodium falciparum*-infected symptomatic patients aged > six months were enrolled in Kobeni and Timbedra in southern Mauritania in September to October 2013. Co-formulated artesunate-amodiaquine was administered at the recommended dose over three days. Patients were followed until day 28. Parasitological and clinical response was classified according to the standard 2009 World Health Organization protocol.

**Results:**

A total of 130 patients (65 in Kobeni and 65 in Timbedra) were enrolled in the study. Seventeen patients (13.1%) were either excluded (before PCR correction) or lost to follow-up. Based on the per protocol analysis, artesunate-amodiaquine efficacy (i.e., the proportion of adequate clinical and parasitological response) was 96.6% in Kobeni and 98.2% in Timbedra before PCR correction. Late clinical failure was observed in two patients in Kobeni and one patient in Timbedra. After PCR correction, the efficacy rate in the two study sites was 98.2%. On day 3, all patients were afebrile and had negative smears. Treatment was well tolerated.

**Conclusions:**

Artesunate-amodiaquine is well tolerated and highly efficacious for the treatment of uncomplicated *P. falciparum* malaria. In the majority of patients, fever and parasitaemia were rapidly cleared before day 3. The results support the national anti-malarial drug guideline for a continued use of artesunate-amodiaquine as a first-line drug for uncomplicated malaria in southern Mauritania.

## Background

Malaria is one of the major public health problems in Mauritania. Every year, between 200,000 and 300,000 malaria cases are officially notified, mostly without laboratory confirmation of clinical diagnosis [1]. Most *Plasmodium falciparum* infections occur in the sahelian south of the country, where transmission is seasonal (generally July to October or November), and malaria represents the first cause of outpatient consultation (25%), hospitalization (35.5%) and mortality (39%).

Resistance to chloroquine and sulphadoxine-pyrimethamine is widespread in West Africa [2]. Since about a decade ago, many West African countries have initiated the evaluation of therapeutic efficacy of artemisinin-based combination therapy (ACT), in particular artesunate-amodiaquine (ASAQ) and artemether-lumefantrine, for the treatment of acute uncomplicated falciparum malaria, and have adopted new treatment guidelines based on the systematic use of ACT [2].

In Mauritania, clinical data on anti-malarial drug efficacy are scarce. In one of the first published papers on a regional survey of clinical resistance to chloroquine between 1987 and 1990 in francophone West Africa, it was reported that Mauritania was still free of chloroquine resistance [3]. However, in a clinical study performed in 1994 in Kiffa, southern Mauritania, using the seven-day test, three of 31 (9.7%) patients still had positive smears and one remained febrile on day 7 [4]. In 1997, indirect evidence for chloroquine resistance *in vitro* was reported in thre of five *P. falciparum* isolates obtained from travellers returning from Mauritania to France [5]. Chloroquine-resistant *P. falciparum* was confirmed in another clinical study conducted in 1998 in Aioun and Kobeni (southern Mauritania) in which it was reported that 33 of 85 (38.8%) patients treated with chloroquine and followed for 14 days failed to respond to the treatment [6]. The high prevalence of mutant *P. falciparum* chloroquine resistance transporter (*pfcrt*) found in that study supports clinical resistance to chloroquine in southern Mauritania. By contrast, analysis of key codons in the molecular markers for sulphadoxine-pyrimethamine resistance (dihydropteroate synthase (*dhps*) and dihydrofolate reductase (*dhfr*), respectively) in the same samples suggested low prevalence (<22%) of antifolate resistance [7].

Since 2006, in line with the anti-malarial drug policies in neighbouring countries, notably Senegal and Mali, Mauritanian Ministry of Health recommends the systematic use of ASAQ and artemether-lumefantrine for the first- and second-line treatment of acute uncomplicated malaria, respectively. This new treatment guideline is not based on data obtained in the country as ACT has never been evaluated before in Mauritanian patients using a standardized protocol. The present single-arm study was performed to evaluate the efficacy and tolerance of ASAQ in *P. falciparum*-infected, symptomatic paediatric and adult patients in southern Mauritania where *P. falciparum* is known to be endemic.

## Methods

### Study sites

**Figure 1:**
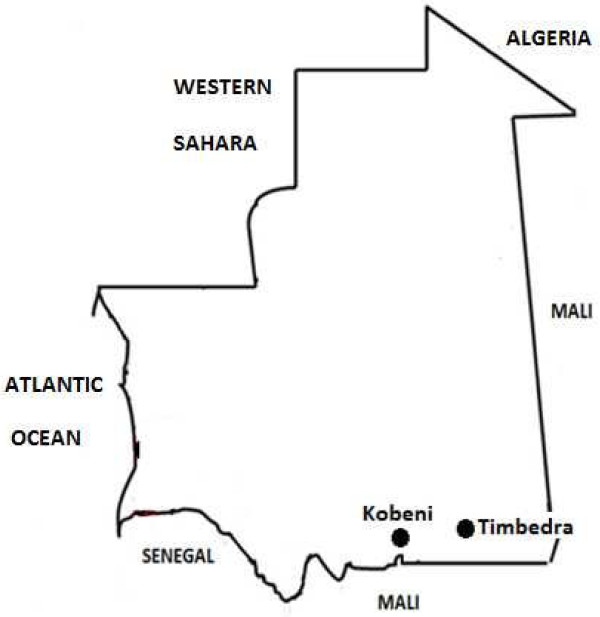
**Study sites in Mauritania.**

The clinical studies were conducted in two study areas in southern Mauritania: Kobeni (Hodh El Gharbi province) and Timbedra (Hodh Echargui province) (Figure 1). The town of Kobeni (15°49′02″ north, 09°24′39″ west) is situated about 105 km south of the city of Aioun (regional capital of Hodh El Gharbi, 980 km southeast of Nouakchott) along Aioun-Nioro route and lies about 20 km from the border with Mali. According to the Mauritanian Office Nationale de la Statistique, there were 97,239 inhabitants in the department (locally referred to as *moughataa*) of Kobeni in 2013. Only one health centre attends to the health needs of the population residing in Kobeni.

The city of Timbedra (15°49′02″ north, 09°24′39″ west) is situated in the southeastern region of the country (Hodh Ech Chargui region) and lies along the major road, *la route de l’Espoir*, about 1,100 km from Nouakchott. There were 76,932 inhabitants in the department (*moughataa*) of Timbedra in 2013. The agricultural and pastoral lifestyle in these two cities is similar. During the rainy season (July to October or November), an average of 30 febrile cases per day are treated in the health centres in Kobeni and Timbedra.

### Patients

Patients spontaneously consulting at the health centres in September and October 2013 were enrolled after informed written consent if the following criteria were met: age > six months old (without upper limit), *P. falciparum* mono-infection with parasitaemia between 250 and 100,000 asexual parasites/μL, presence of fever (axillary temperature ≥37.5°C) or history of fever 24 hours preceding medical consultation, and ability to swallow oral medication [8]. Patients with danger signs or signs of severe and complicated malaria, as defined by the World Health Organization (WHO) [9], mixed *Plasmodium* species, severe malnutrition, fever due to concomitant diseases, and history of allergic reaction to amodiaquine or artemisinin derivatives were excluded. Due to the relative contra-indication of artemisinin derivatives during the first trimester of pregnancy and during breastfeeding, married women with a positive pregnancy test and breastfeeding women were excluded from the present study. In addition, due to the ethical problem in performing pregnancy test in adolescent girls aged between 12 and 18 years old and single adult women >18 years old, these two categories of patients were excluded from the study protocol.

### Laboratory examinations, treatment and follow-up

Finger-prick capillary blood was collected to prepare and examine Giemsa-stained thin and thick smears under the microscope, according to standard WHO procedures [10]. Blood samples were imbibed onto Whatman 3MM filter paper (GE Healthcare Life Sciences, Bucks, UK) and dried to store parasite DNA. Haemoglobin levels were measured using HemoCue^®^ Hb 301 system (HemoCue AB, Angelholm, Sweden).

ASAQ (batch no. 1084 for paediatric tablet containing 25 mg artesunate + 67.5 mg amodiaquine base; batch no. 5433 for adult tablet containing 100 mg artesunate + 270 mg amodiaquine base; Sanofi-Aventis, Antony, France) was administered on days 0, 1 and 2 at the doses recommended by the manufacturer on mg base per weight basis. Each dose administration was supervised by one of the team members, and patients were observed for at least 30 minutes after drug intake for possible vomiting or adverse effects. In case of vomiting during the observation period, the same dose was given, and the patient was observed for an additional 30 minutes. In case of repeated vomiting, the patient was withdrawn from the study protocol and rescue treatment was administered (parenteral quinine, 8 mg base/kg body weight three times a day for seven days or quinine followed by ASAQ or artemether-lumefantrine as soon as the patient tolerated oral medication). Paracetamol (10 mg/kg body weight) was administered to all patients three times a day for fever and headache.

Patients were followed on days 1, 2, 3, 7, 14, 21, and 28 [8]. Finger-prick capillary blood was collected for blood examination from day 2 to day 28 and imbibed onto Whatman 3MM filter paper from day 7 to 28, including any unscheduled visit due to an occurrence of fever, clinical aggravation, or treatment failure. Patients who failed to respond to ASAQ treatment were treated with artemether-lumefantrine (20 mg of artemether + 120 mg of lumefantrine per tablet; one to four tablets per dose, twice a day for three days, according to the manufacturer’s instructions), as recommended by the national guideline for anti-malarial treatment. In Kobeni, haemoglobin measurement was performed on day 0 and repeated on day 28.

### Study endpoints

The primary endpoint was determined on day 28 and classified into one of the following categories: early treatment failure (ETF), late clinical failure (LCF), late parasitological failure (LPF), or adequate clinical and parasitological response (ACPR), as defined by the WHO [8]. Treatment failure refers to one of the following outcomes: ETF, LCF or LPF. ACPR is synonymous with treatment success. The secondary endpoints included the proportion of afebrile patients with negative smears on day 3, and for the study conducted in Kobeni, haemoglobin improvement on day 28.

### PCR and genotyping

Parasite DNA was extracted from filter papers using the Chelex method [11]. *Plasmodium* species was determined using 18S rRNA as the target molecule [12]. Genotypes of paired pre-treatment and recurrent parasites on or after day 7 were determined to distinguish between recrudescence (i.e., re-appearance of the same parasite population as that of pretreatment sample) and re-infection (i.e., appearance of different parasite populations). Genotyping was based on three polymorphic markers, merozoite surface antigen-1 (*msa1*), merozoite surface antigen-2 (*msa2*), and glutamine-rich protein (*glurp*), as recommended by the WHO [13].

### Statistical analysis

The sample size of 50 patients (plus 20% to anticipate losses due to exclusions, withdrawals and lost-to-follow-up), i.e., a minimum of 60 patients, was calculated on the basis of an expected failure rate of 5% extrapolated from data obtained in Senegal, 10% precision and a confidence level of 95% [8].

Haemoglobin values on day 0 and day 28 were compared using the paired *t*-test. Proportions were arranged in a 2 × 2 contingency table and compared using Fisher’s exact test. All statistical tests were two-sided, and the level of statistical significance was fixed at *P* <0.05. SigmaStat 3.5 (Systat Software, Inc, Point Richmond, CA, USA) software was used for these analyses.

As recommended in the WHO protocol [8], per protocol analysis of the percentage of ACPR and Kaplan-Meier survival analysis were performed to calculate the probability of the time to treatment failure during the 28-day follow-up period. Data were entered into the standard pre-programmed Excel worksheet provided by the Global Malaria Programme, WHO, for per protocol analysis. Survival curves were plotted and analysed using Prism 4.0 (GraphPad Software, Inc, La Jolla, CA, USA) software. Quantitative variables were compared between the patient population in Kobeni and Timbedra using the unpaired *t*-test. The significance level was fixed at *P* <0.05.

### Ethical approval

The study was reviewed and approved by an *ad hoc* Mauritanian national ethics committee and the ethics committee of the WHO (Geneva, Switzerland). The purpose of the study was explained to the patients (and their parents or legal guardians for children and adolescents) in local dialect. Informed written consent was obtained from adult patients or caretakers of paediatric patients aged under 12 years. For adolescents aged between 12 and 18 years old, an informed written consent was obtained from both the patients themselves and their parents or legal guardians.

## Results

A total of 130 patients (65 patients in each study site) were enrolled in the study (Table 1). There was no significant difference (*P* >0.05) in the mean age and body weight of the two patient populations in Kobeni and Timbedra. However, the mean body temperature and parasitaemia were significantly higher (*P* <0.05) in Kobeni than in Timbedra. This observation was probably related to the fact that the majority of patients seen in Kobeni health centre (53 of 65 patients, 81.5%) were febrile at the time of consultation, whereas in Timbedra most patients (30 of 65 patients, 46.1%) presented with a recent history of fever but were afebrile at the time of consultation and enrollment.

**Table 1:** **Patient characteristics on inclusion in two study sites in southern Mauritania**

Characteristics	Kobeni	Timbedra
Number of enrolled patients	65	65
Mean age (±SD, range), yr	10.5 ± 5.7 (2–30)	11.9 ± 11.2 (1–65)
Age groups		
Under 5 years old, n (%)	7 (10.8)	11 (16.9)
5-18 years old, n (%)	51 (78.5)	48 (73.8)
>18 years old, n (%)	7 (10.8)	6 (9.2)
Sex ratio (M/F)	1.3 (37/28)	1.5 (39/26)
Mean weight (±SD, range), kg	29.4 ± 15.0 (8–70)	29.0 ± 17.9 (7–0)
Mean body temperature (±SD, range), °C	38.6 ± 1.0 (36.6-40.6)*	37.6 ± 1.3 (36.0-40.6)
Proportion of patients with fever (≥37.5°C) (%)	81.5	46.1
Geometric mean parasitaemia (95% confidence interval, range), asexual parasites/μL	9,230 (7,050-12,100)*	2,090 (1,660-2,630)
	1,860-100,400	312–22,600

*Asterisks denote a statistically significant difference (*P* <0.05) in the mean values between the patient populations in Kobeni and Timbedra.

Of 130 enrolled patients, nine (6.9%) were excluded after inclusion for protocol violation (n = 3), repeated vomiting (n = 3), mixed *P. falciparum*-*Plasmodium vivax* infection (n = 1), or withdrawal of consent (n = 2), and eight (6.2%) were lost to follow-up (Table 2). The per protocol population before PCR correction consisted of 58 and 55 patients in Kobeni and Timbedra, respectively.

**Table 2:** **Per protocol analysis of clinical outcome of artesunate-amodiaquine treatment in southern Mauritania**

Outcome	Kobeni	Timbedra
Number of enrolled patients	65	65
Exclusion after enrollment (before PCR correction), n	7	2
Lost-to-follow-up, n	0	8
Per protocol population (before PCR correction), n	58	55
PCR-uncorrected outcome		
Early treatment failure, n (%)	0	0
Late clinical failure (LCF), n (%)	2 (3.4)	1 (1.8)
Late parasitological failure, n (%)	0	0
Adequate clinical and parasitological response, n (%)	56 (96.6)	54 (98.2)
Per protocol population after PCR correction, n*	57	55
PCR-corrected outcome		
Early treatment failure, n (%)	0	0
Late clinical failure (LCF) due to recrudescence, n (%)	1 (1.8)	1 (1.8)
Late parasitological failure, n (%)	0	0
Adequate clinical and parasitological response, n (%)	56 (98.2)	54 (98.2)
Patients with fever on day 1, n (%)	13 (22.4)	7 (12.7)
Patients with fever on day 2, n (%)	2 (3.4)	0
Patients with fever on day 3, n (%)	0	0
Patients with parasitaemia on day 2, n (%)	46 (79.3)	25 (45.5)
Patients with parasitaemia on day 3, n (%)	0	0

*In Kobeni, one patient was excluded (i.e., censored) after PCR correction due to re-infection.

In Kobeni, per protocol analysis showed ASAQ efficacy of 96.6% before PCR correction. There were two cases of LCF, one occurring on day 5 and the other on day 27. The patient responding with LCF on day 5 (i.e., recrudescence) was a 20-month-old boy presenting with a high-grade fever (39.8°C), a parasitaemia of 17,890 asexual parasites/μL, and haemoglobin of 7.8 g/dL on day 0. He was afebrile from day 1 to day 3 and had a positive smear on day 2 (4,600 asexual parasites/μL) and a negative smear on day 3, but was seen on day 5 with a high-grade fever of 39.6°C and a positive smear (880 asexual parasites/μL). There were no signs and symptoms of clinical aggravation. The patient was treated with artemether-lumefantrine. The other patient responding with LCF was an eight-year-old boy presenting with a high-grade fever of 40.2°C and 17,140 asexual parasites/μL on day 0. He initially responded well to ASAQ treatment. He was afebrile from day 1 to day 21 and had a parasitaemia of 460 asexual parasites/μL on day 2 and negative smears from day 3 to day 21. On day 27, the patient was febrile (37.6°C) and had a positive smear (13,620 asexual parasites/μL). PCR analysis showed that the recurrence of fever and positive smear on day 27 was due to re-infection. After PCR adjustment, ASAQ efficacy was 98.2% in Kobeni.

In Timbedra, the PCR-unadjusted outcome also showed high efficacy (98.2% ACPR) in the per protocol population, with only one LCF occurring on day 21 in a seven-year-old boy. This patient presented with fever (38.2°C) and 1,782 asexual parasites/μL on day 0. He was afebrile from day 1 to day 14 and aparasitaemic from day 2 to day 14. On day 21, the patient was seen with a low-grade fever (37.7°C) and 432 asexual parasites/μL. PCR analysis showed recrudescence.

**Figure 2:**
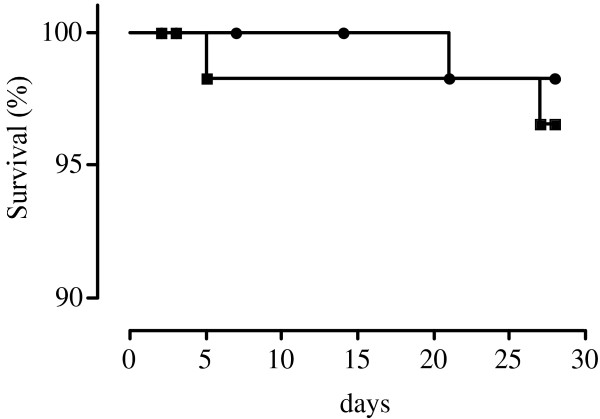
**Survival curves after artesunate-amodiaquine treatment (PCR-uncorrected) in Kobeni (black squares) and Timbédra (black circles).**

**Figure 3:**
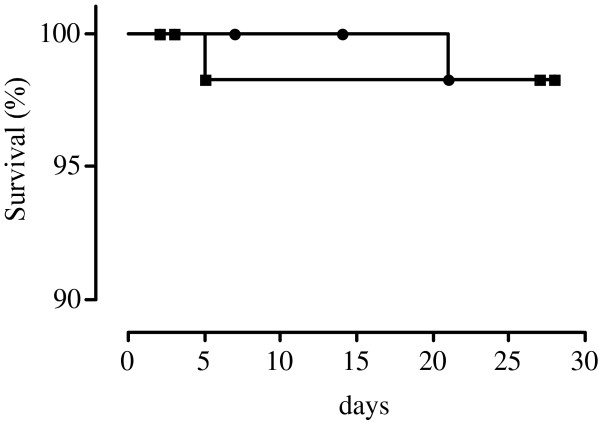
**Survival curves after artesunate-amodiaquine treatment (PCR-corrected) in Kobeni (black squares) and Timbédra (black circles).**

The overall efficacy of ASAQ was 98.2% after PCR correction (56 of 57 patients with ACPR (one patient was censored due to re-infection) in Kobeni and 54 of 55 patients with ACPR in Timbedra) (Figures 2 and 3). In both study sites, there was no case of LPF or ETF. ASAQ cleared fever and parasitaemia rapidly in Mauritanian patients (Table 2). Per protocol analysis showed that 13 and seven patients were still febrile on day 1 and 2 and no patients were febrile on day 2 in Kobeni and Timbedra, respectively. None of the patients was febrile or parasitaemic on day 3.

ASAQ was well-tolerated. Thirty-three of 130 patients (25.4%) reported the following mild and transient adverse effects (n, %): nausea or vomiting (19, 14.6%), headache (9, 6.9%), dizziness (1, 0.8%), anorexia (1, 0.8%), pruritus (2, 1.5%; one patient reported pruritus on day 1 and second patient on day 3), and palpitation (1, 0.8%). Three patients with LCF (two in Kobeni and one in Timbedra) did not report vomiting or any other adverse effects. Although it is difficult to distinguish between malaria-associated symptoms and drug-induced adverse effects, the adverse events reported by the patients resolved spontaneously within 24 hours following administration of the first treatment dose. Pruritus that occurred on day 3 in one patient disappeared on day 4 without any specific treatment. No serious adverse effect was observed.

Paired haemoglobin values were available from all 56 patients followed until day 28 in Kobeni. There was a statistically significant (*P* <0.05) increase in the mean (± SD) haemoglobin value from 9.94 ± 1.66 g/dl on day 0 to the mean (± SD) haemoglobin value of 11.2 ± 1.5 g/dl on day 28. The improvement in haemoglobin values is another benefit of a rapid and effective anti-malarial treatment for patients, in particular in young African children who tend to suffer from malaria-associated anaemia more than adults.

## Conclusions

The rapid and high efficacy of ASAQ observed in the present study is comparable to that found in the neighbouring countries, Senegal and Mali [14–16]. The results of the first clinical evaluation of ASAQ efficacy in Mauritania also showed that this ACT is well tolerated by malaria-infected Mauritanian children and adults. Fever and parasitaemia were rapidly cleared before day 3 in the majority of patients. Treatment was associated with an improvement in haemoglobin values on day 28. These clinical data support the current treatment guideline that recommends the use of ASAQ for the first-line treatment of acute uncomplicated falciparum malaria in southern Mauritania.

## Abbreviations

ACPR: Adequate clinical and parasitological response
ACT: Artemisinin-based combination therapy
ASAQ: Artesunate-amodiaquine
*dhps*: Dihydropteroate synthase
*dhfr*: Dihydrofolate reductase
ETF: Early treatment failure
LCF: Late clinical failure
LPF: Late parasitological failure
WHO: World Health Organization.
